# Environmental associations with gene transcription in Babine Lake rainbow trout: evidence for local adaptation

**DOI:** 10.1002/ece3.531

**Published:** 2013-03-19

**Authors:** Kyle W Wellband, Daniel D Heath

**Affiliations:** 1 Great Lakes Institute for Environmental Research, University of WindsorWindsor, Ontario, Canada; 2 Department of Biological Sciences, University of WindsorWindsor, Ontario, Canada

**Keywords:** Co-inertia analysis, gene expression, qPCR, salmonid

## Abstract

The molecular genetic mechanisms facilitating local adaptation in salmonids continue to be poorly characterized. Gene transcription is a highly regulated step in the expression of a phenotype and it has been shown to respond to selection and thus may be one mechanism that facilitates the development of local adaptation. Advances in molecular genetic tools and an increased understanding of the functional roles of specific genes allow us to test hypotheses concerning the role of variable environments in shaping transcription at known-function candidate loci. To address these hypotheses, wild rainbow trout were collected in their first summer and subjected to metabolic and immune challenges. We assayed gene transcription at candidate loci that play a role in the molecular genetic response to these stresses, and correlated transcription with temperature data from the streams and the abundance and diversity of bacteria as characterized by massively parallel pyrosequencing. Patterns of transcriptional regulation from resting to induced levels varied among populations for both treatments. Co-inertia analysis demonstrated significant associations between resting levels of metabolic gene transcription and thermal regime (*R*^2^ = 0.19, *P* = 0.013) as well as in response to challenge (*R*^2^ = 0.39, *P* = 0.001) and resting state and challenged levels of cytokine gene transcription with relative abundances of bacteria (resting: *R*^2^ = 0.25, *P* = 0.009, challenged: *R*^2^ = 0.65, *P* = 0.001). These results show that variable environments, even within a small geographic range (<250 km), can drive divergent selection among populations for transcription of genes related to surviving stress.

## Introduction

Local adaptation is characterized by local genotype advantage where individuals experience higher fitness in their local environment than any other environment in which they could exist (Kawecki and Ebert 2004). Local adaptation implies that local environmental forces have acted, via natural selection, to increase traits that are advantageous to individuals in that environment. In salmonids, local adaptation is facilitated by high levels of natal philopatry and population subdivision that occurs across a landscape of variable environments (Quinn 2005). The scale and extent to which local adaptation occurs in salmonid populations appears to be context- and trait dependent (Fraser et al. 2011) and is affected by the complex interactions of selection and drift within populations, and gene flow among populations. However, local adaptation is primarily thought to be a response to environmental variation, and indeed recent reviews have highlighted the roles temperature and diseases play in determining functional divergence among populations (Garcia de Leaniz et al. 2007; Fraser et al. 2011). A better understanding of the patterns and processes that affect the development and maintenance of local adaptation is critical to our understanding of speciation processes (Schluter 2000) as well as the effective conservation of locally adapted populations (Fraser and Bernatchez 2001).

Despite the use of quantitative genetics to study the genetic architecture of local adaptation, (e.g., Aykanat et al. 2012a) the molecular genetic mechanisms of local adaptation are currently not well characterized. Several indirect methods for exploring the genetic mechanisms contributing to local adaptation have been developed, including comparisons of population divergence at functional versus neutral loci (*Q*_ST_ vs. *F*_ST_) and correlations of environmental variables or gradients with phenotypic/genetic traits (reviewed by Fraser et al. 2011). However, more direct approaches have become feasible with the advent of rapid, cost-effective gene transcription assay methods. Measures of gene transcription have recently been shown to be powerful tools to investigate the molecular genetic nature of local adaptation because transcription: (1) is a heritable phenotype and (2) has direct consequences for an organism's growth, development, and response to stimuli (Fay and Wittkopp 2008). Gene transcription profiles have been used to characterize the mechanisms of local adaptation in a variety of ways. Parallel evolution of transcription profiles has been demonstrated among sympatric whitefish species pairs (Derome et al. 2006; St-Cyr et al. 2008). Breakdown of gene transcription among wild-farmed hybrids have been shown in Atlantic salmon (Normandeau et al. 2009; Tymchuk et al. 2010). Gene transcription profiles have also been linked to fitness of wild Sockeye salmon experiencing a changing environment (Miller et al. 2011) and targeted studies of candidate loci transcription have also had success in detecting signatures of rapid evolution in natural populations (e.g., Jeukens et al. 2009; Aykanat et al. 2011).

The utility of gene transcription for identifying differences among populations is clear; however, few studies have been able to attribute divergence among populations to specific local environmental variation. Selective forces influencing phenotypic variation in salmonid populations may include biotic and/or abiotic components of the environment (Taylor 1991; Garcia de Leaniz et al. 2007). Abiotic conditions associated with stream size (e.g., water temperature, flow, etc.) are important in explaining among-group phenotypic variation (reviewed by Garcia de Leaniz et al. 2007). Salmonid populations persist under a wide range of stream temperatures (e.g., Elliot et al. 1998) some of which are near critical thermal maxima for these species during summer. Transcription of genes that underlie the metabolic demands associated with survival under temperature stress are thus a potentially locally adaptable trait in situations where temperature regimes differ among populations. The primary response to metabolic stress in fish involves stimulation of the hypothalamus-pituitary-interrenal axis resulting in the release of glucocorticoids such as cortisol (Mommsen et al. 1999) and cortisol levels are heritable, can be differentially selected for, and have consequences for fitness (Fevolden et al. 2002). In salmonids, cortisol release has been shown to trigger a reorganization of metabolism in the liver that is mediated by gene transcription, and that facilitates the rapid deployment of glucose to tissues providing the fuel to regain homeostasis (Wiseman et al. 2007).

The role of disease in driving salmonid local adaptation is also well established. Both resistance and susceptibility to a variety of bacterial and parasitic infections have been associated with certain major histocompatibility (MH) alleles (e.g., Wynne et al. 2007: amoebic gill disease; Turner et al. 2007: bacterial kidney disease; Glover et al. 2007: sea lice; Dionne et al. 2009: myxozoa). Many of those studies were conducted under laboratory conditions in response to a single challenge. In contrast, MH heterozygosity has been associated with resistance to infection in salmon experiencing a complex bacterial community, despite no single allele alone conferring resistance (Evans and Neff 2009). Evidence of selection at a variety of immune-related loci has been demonstrated in natural populations (Tonteri et al. 2010), reinforcing the importance of studying immune system evolution under natural conditions. Few studies have characterized stream pathogen communities among natural salmon populations, and relatively little is known about the spatial and temporal patterns of abundance of fish pathogens (McVicar et al. 2006). However, in general, microbial stream communities in temperate regions have stronger spatial structuring compared with temporal structuring despite the seasonal trends of succession (e.g., Hullar et al. 2006). The strength and direction of selection on the immune system varies across different life stages of salmon (de Eyto et al. 2011) indicating that if life stage-specific local adaptation to pathogens occurs, much of it would likely be in the first year of life, as juvenile salmonids experience high mortality (up to 90%, Quinn 2005) during this period. Recognition of pathogens and the subsequent immune response is triggered through a complex set of receptors and signaling molecules (Medzhitov and Janeway 1997). The critical components of those pathways are small signaling proteins, cytokines, and chemokines, which direct how the immune system responds to pathogens (Secombes et al. 1996; Bird et al. 2006). Transcriptional control of cytokine and chemokine activity has been documented in various tissues in fish (Scapigliati et al. 2006; Raida and Buchmann 2008), thus selection has the potential to act upon transcription of these signaling molecules.

The environmental factors expected to drive selection among habitats, coupled with our understanding of gene function, makes it possible to select candidate genes to test for specific functional divergence based on environmental variation among putatively locally adapted populations. Here, we test the hypothesis that gene transcription at candidate loci differs among genetically structured populations, and that attributes of the local environment are correlated with gene transcriptional profiles. Specifically, we investigate the role that temperature and bacterial community diversity play in determining gene transcription variation at biologically relevant loci among naturally occurring rainbow trout populations from Babine Lake, British Columbia. We use real-time quantitative polymerase chain reaction (RT-qPCR) to quantify gene transcription combined with novel next-generation pyrosequencing to quantify bacterial community diversity and 1 year of temperature data to provide evidence that local environments drive transcriptional difference, and ultimately, the evolution of local populations. This work provides insight into the mechanisms controlling local adaptation of salmon populations, with implications for how we view adaptation and the management of this species.

## Methods

### Sampling sites and protocol

**Figure 1:**
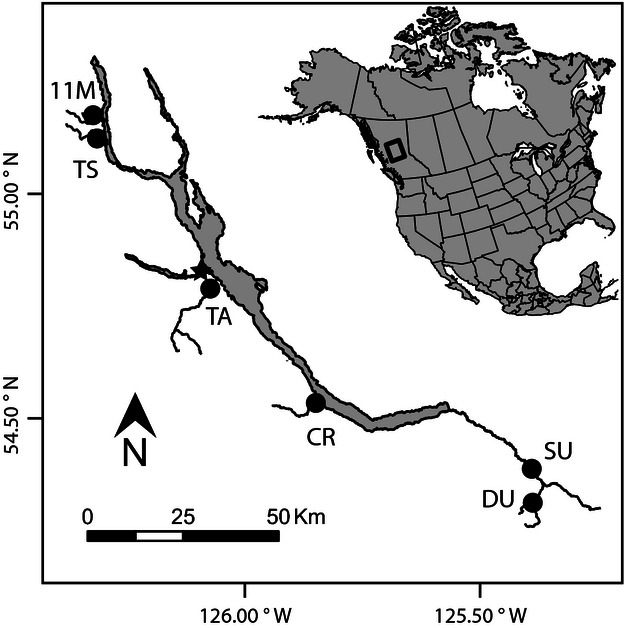
Map of Babine Lake and the tributaries sampled (closed circles) for juvenile rainbow trout. The solid star indicates the Fulton River facility where the fish were held and experiments were conducted.

We sampled six tributaries of Babine Lake (Fig. 1) known to have rainbow trout spawning populations (Bustard 1989). In Babine Lake, rainbow trout spawn in over 34 tributaries from late May and early June, fry emerge from the gravel during mid-July to the first week of August and rear for up to 3 years in the stream before descending to the lake to spend their life as adults (Bustard 1989). Tributaries were chosen to represent a range of environmental conditions and watersheds, as well as geographic distances from one another (Bustard 1989; Koehler 2010). Tsak (TS) and 11 Mile (11M) creeks are small tributaries at the northern end of Babine Lake, Tachek (TA) and Cross (CR) creeks are medium and small tributaries, respectively, located near the midpoint of the lake, and Duncan (DU) creek and the Sutherland River (SU) are small and large tributaries, respectively, which drain a large watershed at the southern end of Babine Lake (Fig. 1, Table 1). Stream temperature in late August of 2010 at the time of fish collection ranged from a high of 11.5°C in DU creek to 8.5°C in 11M creek and followed a decreasing trend with increasing latitude (SU = 11.0°C, CR = 10.0°C, TA = N/A, TS = 9.0°C). The temperature logger from TA Creek was not recovered and resulted in no temperature data for this tributary. Genetic population structure has been demonstrated among all the tributaries we sampled (Koehler 2010) indicating reduced gene flow and the potential for the evolution of adaptive divergence.

**Table 1:** Temperature profile and eubacterial community characterization of six Babine Lake tributaries ordered from north to south along the axis of the lake

Tributary	Abbr	Lat (°N)	Long (°W)	FL (mm)	MT (°C)	MDR (°C)	ADR (°C)	DA5 (m/dd)	Area (Km^2^)	All 16S	16S genus
11 Mile	11M	55.17806	126.62614	39–53	13.5	4.0	1.2	5/26	36	1028	324
Tsak	TS	55.13884	126.61987	39–59	14.5	4.0	1.0	5/26	24	621	181
Tachek	TA	54.78710	126.12808	32–64	NA	NA	NA	NA	105	3925	1020
Cross	CR	54.51376	125.70652	36–59	10.5	2.0	1.0	5/26	39	1942	476
Sutherland	SU	54.33987	124.83503	37–58	11.5	2.0	0.8	5/21	1310	2358	562
Duncan	DU	54.26835	124.84741	40–54	12.5	3.5	1.3	5/22	83	1395	479

Temperature data were unavailable for Tachek Creek due to a lost temperature data logger (Represented by NAs in the table).

Abbr, tributary abbreviation; Lat, latitude (decimal degrees); long, longitude (decimal degrees); FL, fork length range (mm) of sampled rainbow trout fry; MT, maximum temperature (°C); MDR, maximum daily temperature range (°C); ADR, average daily temperature range (°C); DA5, first day with average temperature above 5°C; Area, watershed area (Km^2^); All 16S, total number of 16S rRNA sequences per library/tributary; 16S genus, number of 16S rRNA sequences identified to genus per library/tributary.

Approximately 50 young-of-the-year (32–64 mm) rainbow trout (*Oncorhynchus mykiss* Walbaum) were collected from each tributary (23–25 August 2010) by dip netting and electroshocking (Smith-Root BP-15 backpack shocker, Smith-Root, Inc., Vancouver, WA). Fish were placed into heavy plastic bags (60 × 120 cm) containing ambient water from their tributary. The bags were twisted closed to remove any ambient air and oxygen was then bubbled into the water and allowed to accumulate until it filled approximately one-fourth the volume of water in the bag. Bags were sealed and transported (2–6 h) on ice to the Department of Fisheries and Oceans' Fulton River Spawning Channel facility. Fish from each tributary were held in separate cages in a 3 m round tank with water flow-through from Fulton Lake (15 ± 0.5°C). Fish were held for 5 days under starvation to acclimate to hatchery conditions and recover from the capture and transportation stress. Mortality only occurred for several individuals from one population (TA Creek). It is believed that these individuals were chronically stressed prior to sampling due to the presence of many dead fish at the TA Creek sampling location.

### Experimental protocol

Immune and temperature challenges were conducted on a subset of 10 fish from each population. For the immune challenge, fish were randomly selected from each tributary sample and transferred to a 10% Vibrogen 2 vaccine bath containing formalin-inactivated cultures of *Vibrio anguillarum* serotypes I and II and *Vibrio ordalii* (Novartis Animal Health, Mississauga, Ontario, Canada) for 1 min. This protocol has been previously shown to elicit an immune response in Chinook salmon fry, *Oncorhynchus tshawytscha* (Aykanat et al. 2012b). The temperature challenge consisted of randomly selecting a different subset of fry from each tributary and placing them in a water bath of 20 ± 0.5°C water for 1 h. The water temperature was chosen to be 5°C above the ambient temperature of hatchery water, but below the thermal maximum for rainbow trout. Following both treatments, fry were returned to separate cages in the holding tank where they were allowed to recover for 24 h. Control fish were sampled directly from the holding tank prior to any challenge to provide resting state transcription samples. Sampling of tissues occurred for control groups prior to exposure and for challenged groups, 24 h post-exposure. In this design, the transcription of fish sampled 24 h post-exposure will reflect both handling stress as well as the applied treatment; however, the genes we chose to assay are not likely to have high sensitivity to the gentle handling procedure we used 24 h prior to sampling. All fish were humanely euthanized using an overdose solution of clove oil (250 ppm) and gill tissues were dissected, immediately preserved in RNA later (Invitrogen, Burlington, ON, Canada), and stored at 4°C. Samples were frozen at −20°C within 5 days and stored at that temperature until further analysis. Gill tissue was chosen because it is a metabolically important tissue as the primary site of gas and ion exchange as well as it experiences direct exposure to the environment (temperature and pathogens).

### Selection of candidate loci

Wiseman et al. (2007) identified several differentially regulated genes in rainbow trout liver during metabolic stress (*cathepsin D,* glucocorticoid receptor [*GR*], pyruvate kinase [*PK*], and phosphoenolpyruvate carboxykinase [*PEPCK*]). We chose genes to represent rate-limiting, and thus regulatory, steps of major metabolic pathways that are important in all tissues. The function of *PEPCK* and *PK* were inferred from studies across all levels of life and are widely accepted as rate-limiting steps of gluconeogenesis and glycolysis pathways (Pilkis and Granner 1992). Cathepsins are a class of proteolytic enzymes involved in protein degradation pathways where *cathepsin D* is the primary cathepsin responsible for intracellular protein degradation in lysosomes (Fusek and Větvička 2005). *GRs* are central to the activation of a stress response through cortisol signaling and have been widely studied in fish, including rainbow trout (Aluru and Vijayan 2009).

Raida and Buchmann (2008, 2009) identified cytokine/chemokines as significantly up-regulated following immune challenges in rainbow trout (interleukin 1β [*IL-1β*], interleukin 8 [*CXCL-8*], interferon γ [*IFNγ*], tumor necrosis factor α [*TNFα*]), and those genes play important roles in determining downstream responses of the immune system. *IL-1β* and *TNFα* are involved in activating and modulating responses of the immune system by inducing inflammation and altering expression of other cytokines in fish (Whyte 2007). *CXCL-8* is involved in the recruitment of immune effector cells to the sites of infection (Whyte 2007) and *IFNγ* plays important roles in modulating growth, maturation, and differentiation of various immune cells as well as activation of macrophages for killing bacterial and viral pathogens (Robertson 2006). We utilize these genes to assay innate immune response among populations.

### RNA extraction and cDNA synthesis

Total RNA was extracted from gill tissue using mechanical homogenization of tissue in 0.8 mL of TRIZOL (Invitrogen) following the method of Chomczynski and Sacchi (1987). Total RNA preparations were assessed for quality using gel-electrophoresis where clear *28S* and *18S* rRNA bands and minimal low-molecular-weight smear indicated good quality RNA. Purity and concentration of total RNA was assessed using UV spectrophotometry in a Victor 3V plate reader (Perkin Elmer, Waltham, MA). All total RNA preparations had purity values of 1.9–2.1 (A260/A280). Based on the concentration calculated using UV spectrophotometry, total RNA was diluted to 100 ng/μL and treated with DNase 1 (Fermentas, Waltham, MA) to remove genomic DNA contamination. Total RNA was converted to complementary DNA (cDNA) using a High Capacity cDNA kit (Applied Biosystems, Burlington, ON, Canada). Reverse transcriptase (RT) reactions contained 1.0 μg of total RNA, 2 μL of random primers (Applied Biosystems), 4 mmol/L each dNTP, 50 U of MultiScribe RT (Applied Biosystems) and 40 U of RNase Inhibitor (Applied Biosystems) in a 1× RT buffer at a final volume of 20 μL. RT reactions were incubated at 25°C for 10 min followed by 37°C for 2 h and were stopped by incubating at 85°C for 5 min. RNA from DNA–RNA hybrids was degraded using 1 U of RNase H (New England Biolabs, Ipswich, MA) for each RT reaction and incubation at 37°C for 20 min. RT reactions were then diluted to a final volume of 100 μL with ddH_2_O.

### Quantitative real-time PCR

Four biologically relevant genes for each treatment and two reference genes (Table 2) were assayed in six individuals from each population for each treatment. Primers and probes for previously unpublished loci were designed using publicly available cDNA sequences from GenBank (Table 2) and Primer Express software (Applied Biosystems). Where possible, primers were designed across exon–intron boundaries to reduce amplification of residual genomic DNA contamination. Both reference genes have been shown to be stably expressed before and after stress challenges (Ortega et al. 2005; Ching et al. 2010). PCR reactions contained 50 nmol/L Taqman probe, 100 nmol/L forward and reverse primers, and 10 ng of cDNA in a 1× master mix (Taqman Gene Expression master mix, Applied Biosystems). Assays were run in triplicate for reference genes and in duplicate for target genes on an ABI 7500 Real-Time PCR machine (Applied Biosystems) for 45 cycles of 95°C for 30 sec and 60°C for 1 min.

**Table 2:** Primers and probes for quantitative real-time PCR assays of rainbow trout gene transcription for candidate genes chosen for a temperature and immune challenge

Gene	Treatment	Accession	Forward seq	Reverse seq	MGB probe seq	Reference
EF-1α	Reference	AF498320	AATACCCTCCTCTTGGTCGTTTC	CTTGTCGACGGCCTTGATG	TGCGTGACATGAGGC	Aykanat et al. (2011)
ARP	Reference	AY685220	TTGTTTGACTAACTTGCTATTCTTTGC	CGCCGACAATGAAACATTTG	AATTGCTGGATGACTATC	Ortega et al. (2005)
CathepsinD	Temperature	U90321	GGGAGGAACTGACCCGAAGT	GCGGCTGACGTCGAGGTA	CTACAGTGGAGACTTCCA	This paper
GR	Temperature	Z54210	CTGGCTGTTCCTCATGTCGTT	CAACATCCCCCCGTTACACT	CTTGGGCTGGCGCT	This paper
PEPCK	Temperature	AF246149	GCCCCTTCTTCGGCTACAA	CTTGCGGGTCTCCATGCT	TCGGTGACTACCTAGCC	This paper
PK	Temperature	AF246146	TGGGCCGACGATGTAGACA	CCCCTGGCCTTTCCTATGTT	CAGAGTCAACTTCGGC	This paper
IL-1β	Immune	AJ223954; AJ298294	ACATTGCCAACCTCATCATCG	TTGAGCAGGTCCTTGTCCTTG	ATGGAGAGGTTAAAGGGT	Raida and Buchmann (2008)
CXCL-8	Immune	AJ279069	AGAATGTCAGCCAGCCTTGT	TCTCAGACTCATCCCCTCAGT	TTGTGCTCCTGGCCCT	Raida and Buchmann (2008)
IFNγ	Immune	AY795563	CAAACTGGCCCTTAAGTTCCA	TCTGGGCTTGCCGTCTCT	TAAAGAAGGACAACCGCAGG	T. Aykanat, unpubl. data
TNFα	Immune	AJ277604; AJ401377	GGGGACAAACTGTGGACTGA	GAAGTTCTTGCCCTGCTCTG	ACCAATCGACTGACCGAC	Raida and Buchmann (2008)

PCR, polymerase chain reaction; GR, glucocorticoid receptor; PEPCK, phosphoenolpyruvate carboxykinase; PK, pyruvate kinase; IL-1β, interleukin 1β; CXCL-8, interleukin 8; IFNγ, interferon γ; TNFα, tumor necrosis factor α.

PCR efficiency for each amplicon was determined using the program LinRegPCR (Ramakers et al. 2003) and amplicon efficiency, threshold and Cq values were obtained and used to calculate theoretical starting cDNA concentrations (N_0_) per technical replicate in LinRegPCR (Ramakers et al. 2003) using the unbiased method of Tuomi et al. (2010) for hydrolysis probes. Technical replicates for genes were averaged within individuals. Reference genes (*EF-1α* and *ARP*) were combined to create a normalization factor by taking the geometric mean of the N_0_ values for the reference genes within individuals. Transcription of target genes was then expressed as a ratio of the value for the gene relative to the normalization factor (the equivalent of Δ*C*_t_).

### Tributary environment characterization

#### Microbial community

One liter of water was collected from each sampled tributary in May 2011 and filtered through 0.2-μm filters (Pall Life Sciences, Mississauga, ON, Canada). Despite succession of bacterial communities from season to season, stream microbial communities have been shown to have stable spatial structure from year to year (e.g., Hullar et al. 2006). Although our water sampling does not coincide with conditions experienced by the fish sampled for this study, the microbial communities we characterize here provides an estimate of spatial variation and structure of bacteria among the streams we sampled. In addition, the timing of water sampling occurred as rainbow trout were spawning and does in fact represent conditions experienced by rainbow trout eggs every year thus indicating the potential for selection. Environmental DNA (eDNA) was extracted from each sample using a modified phenol:chloroform and CTAB buffer extraction (Chaganti et al. 2012) with 3 freeze–thaw cycles and mechanical homogenization to lyse bacterial cells. A 278 base pair portion of the *16S* ribosomal gene that contains the V6 variable region (for taxonomic identification) was amplified with primers corresponding to 786–1063 bp of the *E. coli 16S* gene (Huws et al. 2007). Polymerase Chain Reactions (PCRs) were performed in a 25 μL volume and contained 10 mmol/L Tris-HCl (pH 8.3), 50 mmol/L KCl, 2.5 mmol/L MgCl_2_, 200 μmol/L each dNTP, 0.4 μmol/L primers, 1 U of AmpliTaq DNA polymerase (Applied Biosystems), and 50–100 ng of eDNA. Reactions were amplified for 25 cycles of 94°C for 30 sec, 56°C for 30 sec, and 72°C for 40 sec. The PCRs were then split and amplified in triplicate for 20 cycles using adaptor-modified primers (Titanium primers for 454 pyrosequencing) following the same conditions. PCR products were gel-purified, standardized with respect to concentration and pooled. Emulsion PCR was completed by Engencore (Columbia, SC) using Titanium sequencing chemistry and sequencing was performed on a Titanium PicoTiterPlate (454 Life Sciences, Branford, CT) in a GS FLX pyrosequencer (454 Life Sciences). Raw pyrosequencing data were processed, primer sequences trimmed, and low-quality sequences removed using the RDP pyrosequencing pipeline (Cole et al. 2008). Processed sequences were then classified using the online RDP naive Bayesian rRNA classifier (Wang et al. 2007) with a conservative confidence threshold of 80% to the level of genus, the finest taxonomic resolution available. We calculated simple diversity measures of the bacteria identified to genus including number of genera and the Shannon diversity index using the software mothur version 1.22.1 (Schloss et al. 2009).

#### Temperature profile

Temperature data loggers (iBCod DS1922L, Maxim Integrated Products, San Jose, CA) were deployed in each tributary during initial sampling (August 2010) and recovered from 5 of the 6 tributaries the following spring (May 2011; the TA Creek data logger was lost over the winter). Data loggers recorded water temperature every 4 h to an accuracy of 0.5°C. Maximum and minimum water temperatures, average and maximum daily range of water temperature, and the first day with average water temperature above 5°C were calculated.

### Data analysis

All analyses were conducted in the statistical software R version 2.14.1 (R Development Core Team 2011). First, we tested for a correlation between gene transcription for each gene assayed and fork length to assess the role body size played in gene transcription. We also tested for a correlation between gene transcription values with geographic order (South to North) to assess potential geographic influences on gene transcription because isolation by distance has been demonstrated for these populations using neutral microsatellite markers (Koehler 2010). To test for transcriptional response to our challenges, we used *T*-tests to test for differences between control and challenged transcription for each gene in each population. To account for multiple tests, we calculated the false discovery rate (FDR) for each challenge and global *P*-values for each locus using 1000 random permutations of the data. FDRs were calculated for each challenge as the random expectation of the number of significant tests per permutation divided by the number of observed significant tests in the original data. To test for population differences in response to stress, we subtracted mean population resting state transcription from challenged individuals. We then compared population responses in a one-way analysis of variance (ANOVA) for each gene.

Due to the number of sites sampled, the number of genes analyzed and the number of environmental parameters considered, the use of simple linear regression to test for relationships among all the variables is not statistically sound. As an alternative, we first used principal components analysis (PCA) to define the major axes of variation among sites in both transcriptional and environmental datasets. We then correlated the major axes of variation in transcription with the major axes of variation in the environmental data using co-inertia analysis (Dolédec and Chessel 1994; Culhane et al. 2003). The co-inertia analysis identified associations of variable loadings among correlated principle components. Statistical significance of these associations was tested using a randomization test where the *P*-value is the probability of the observed associations occurring in 1000 random permutations of the data. We compared transcription PCs under both resting state as well as the differential response to challenge with the relevant environmental PCs. The response to challenge was calculated by subtracting the mean transcription of the control group for each population from each challenged individual from that population. The PCAs were performed separately for transcription of metabolic and immune genes as well as for control and response to challenge groups resulting in a total of four PCAs for the transcriptional data. Separate PCAs were also conducted on the stream temperature profiles and the bacterial community relative abundance. Due to missing stream temperature data, the TA creek samples were omitted from the metabolic gene and stream temperature profile analyses. PCA and co-inertia analyses were conducted in the ade4 package in R (Dray and Dufour 2007).

## Results

### Gene transcription

**Figure 2:**
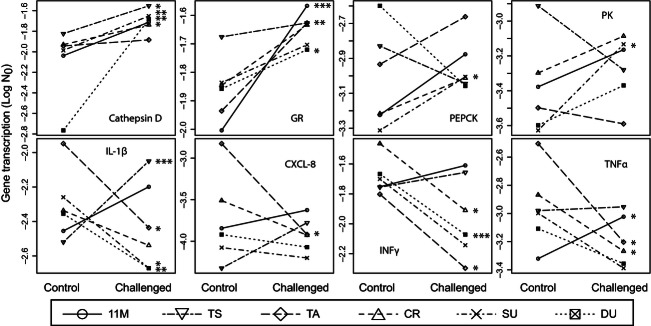
Gene transcription reaction norms for candidate loci from six Babine Lake tributary populations in response to temperature stress (top row) and immune challenge (bottom row). Asterisks indicate significant differences in transcription between control and treatment for each population (*P < 0.05, **P < 0.01, ***P < 0.001).

Response to challenge: All metabolic and immune genes were differentially regulated following challenge in at least one population. *Cathepsin D* and *GR* were up-regulated in multiple populations while *PEPCK* and *PK* only increased significantly in one population (TA and SU, respectively; Fig. 2, Table S1). Of the immune genes assayed *IL-1β, IFNγ,* and *TNFα* transcription was significantly differentially transcribed after challenge in multiple populations and *CXCL-8* only decreased significantly in one population (TA; Fig. 2, Table S1). The only gene that had a significant global response was *IL-1β* (*P* = 0.041). FDRs were calculated to be very low (temperature: FDR = 0.024, immune: FDR = 0.015) indicating that, despite multiple tests, the significance of our results are not obscured by false positives. These results indicate that the challenges we chose do induce transcriptional responses, but that the response is population-specific (Fig. 2, Table S1). To support this argument, results from one-way ANOVAs indicate that population level transcriptional response to challenge differed significantly for all genes assayed (*Cathepsin D: P* = 0.032, *GR, PEPCK, PK, IL-1β, CXCL-8, IFNγ,* and *TNFα* all: *P* < 0.001).

Gene transcription did not correlate with body size (fork length) nor with the order in which sites occur along the axis of the lake for any of the genes or treatments we investigated, suggesting that systematic sampling biases do not influence transcriptional variation. Isolation by distance has been previously demonstrated for the Babine Lake tributary populations using neutral microsatellite markers (Koehler 2010); however, the lack of geographic patterns in our gene transcription data suggests that genetic drift is not driving transcriptional variation among the sampled populations.

### Tributary environment characterization

Eubacterial *16S* rRNA libraries were obtained for all six streams sampled in 2011. Sizes of the trimmed and quality filtered libraries ranged from 621 to 3925 sequences (Table 1). The low overall output of the pyrosequencing is primarily due to the fact that these samples were pooled and run in parallel with many samples for other projects. Our intent was to characterize the common members of the microbial community, those most likely to affect fish health and immune system. The RDP classifier assigned between 181 and 1020 (24–34%) sequences per library to the taxonomic level of genus, the lowest level of classification obtainable. Despite the low overall output of 454 pyrosequencing, we observed considerable microbial diversity both in the number of genera described and in the diversity and relative abundance of those genera (Table 3). Of the sequences assigned to a genus 27–620 (8–61%) belonged to genera that contain at least one species suspected to cause disease in fish. In total, 1174 potentially pathogenic organisms were detected across all *16S* rRNA libraries. *Flavobacterium spp*. accounted for 61% (774 sequences) of all potentially pathogenic organisms detected followed by *Acidovorax spp*. (13%, 150 sequences) and *Corynebacterium spp*. and *Streptococcus spp*. (5%, 57 sequences each). The remaining 16% of sequences were accounted for by 11 genera and their abundances ranged from 1 to 32 individuals detected across all libraries. Overall, the most important potentially pathogenic genus was *Flavobacterium spp*. which ranged in relative abundance from 0 to 50% of the total bacterial community of sampled tributaries (Table 3).

**Table 3:** Bacterial genera richness (Genera), Shannon diversity index (Shannon), and relative abundance (% of total bacterial community) for the four most common genera of suspected fish pathogens from six Babine Lake tributary streams determined using 454 pyrosequencing of 16S rRNA

Tributary	Genera	Shannon	*Acidovorax spp*.	*Corynebacterium spp*.	*Flavobacterium spp*.	*Streptococcus spp*.
11 Mile	42	3.28	0.6	6.6	0	1.1
Tsak	27	1.78	3.4	0.6	2.2	0.3
Tachek	70	2.06	8.1	0	50	0
Cross	51	2.36	5	0	37	0.2
Sutherland	70	3.01	5	5.7	11	0
Duncan	41	3.13	0.6	2.3	4	11.1

Temperature loggers were deployed and successfully recovered from 5 of the 6 tributaries. Data loggers were deployed for 272–285 days spanning a period from late August of 2010 until late May of 2011. Data loggers were deployed in the deepest pools to prevent them from freezing; however, on or about 8 November 2010 all recovered loggers reached low temperatures at 0.0°C and the temperature did not change until the following April. As a result, the average daily range we report includes only the period of time during which water temperatures were recorded to be above 0°C. Maximum recorded temperature, maximum daily range and the average daily range varied among tributaries (Table 1). The first day with a mean daily water temperature above 5°C also varied by as many as 5 days among tributaries, which will impact rainbow trout spawning run timing (Bustard 1990) and egg/fry development rates.

### Transcription–environment associations

PCA of metabolic gene transcription in control and response to challenge groups identified the first two axis of variation that, respectively, explained 76% and 73% of the overall variation in the data (Control: PC1 = 47%, PC2 = 29% and Challenge Response: PC1 = 40%, PC2 = 33%). For the control group, PC1 was loaded primarily by *cathepsin D* and *GR* and PC2 was loaded primarily by *PEPCK*. In the challenge response group, PCA loadings indicated that PC1 was loaded equally by *GR, PEPCK* and *PK* and PC2 was loaded primarily by *cathepsin D*. PCA of the immune gene transcription in control and response to challenged groups each explained 93% or 96% of the variation, respectively, and both identified two major axes of variation in the data (Control: PC1 = 68%, PC2 = 25% and Challenged: PC1 = 69%, PC2 = 27%). Loadings for the immune genes were much more diverse, with three genes contributing approximately equally to PC1 for both experimental groups (*IL-1β, CXCL-8, TNFα*) and one gene loaded onto PC2 (*IFNγ*).

The first two principal components of the stream temperature dataset (maximum stream temperature, maximum daily range, average daily range, and first day with average temperature above 5°C) explained 76% of the variation (PC1 = 47%, PC2 = 29%). The first axis was loaded primarily by maximum temperature, maximum daily range, and to a lesser extent average daily range. The second axis was loaded primarily by the first day with average temperature above 5°C. The bacterial community PCA produced one axis that explained 86% of the variation in bacteria communities among sites. This PC was primarily driven by differences in relative abundance of the genus *Flavobacterium* among tributaries.

**Figure 3:**
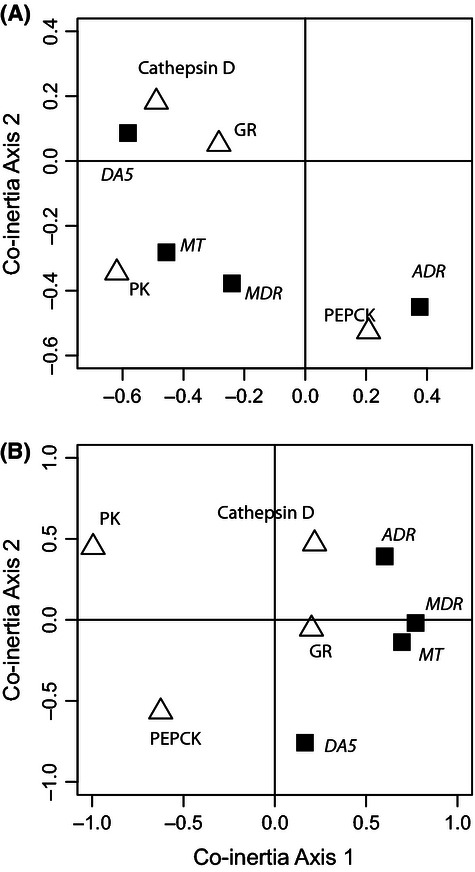
Co-inertia analysis of stream temperature profile (black squares, italic font) for five Babine Lake Tributaries and resting state (A) or response to challenge (B) metabolic gene transcription (open triangles, regular font) for rainbow trout fry taken from the five tributaries. Positive associations indicated by closer than expected proximity in coordinate space and negative associations indicated by greater than expected distances in coordinate space. MT, maximum temperature (°C); MDR, maximum daily temperature range (°C); ADR, average daily temperature range (°C); DA5, first day with average temperature above 5°C; CthpnD, cathepsin D; GR, glucocorticoid receptor; PEPCK, phosphoenolpyruvate carboxykinase; PK, pyruvate kinase.

The co-inertia analysis revealed a significant association between stream temperatures and metabolic gene transcription in the control treatment (*R*^2^ = 0.19, *P* = 0.013). The strongest associations were *PEPCK* transcription with average daily temperature range and *PK* transcription with maximum stream temperatures (Fig. 3A). *Cathepsin D* and *GR* showed moderate negative associations with average daily temperature range. The co-inertia analysis of stream temperatures with response to temperature challenge gene transcription demonstrated even stronger associations (*R*^2^ = 0.39, *P* = 0.001, Fig. 3B).

**Figure 4:**
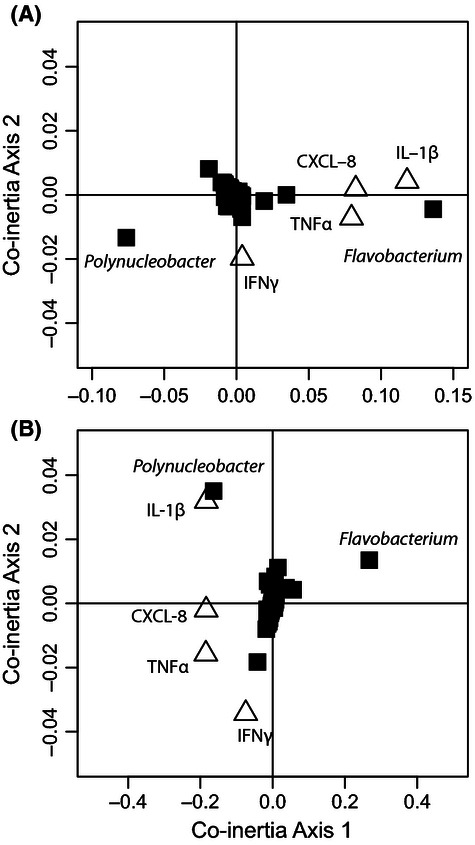
Co-inertia analysis of relative abundance of stream bacteria (black squares, italic font) for six Babine Lake Tributaries and resting state (A) or response to challenge (B) cytokine/chemokine gene transcription (open triangles, regular font) for rainbow trout fry taken from the six tributaries. Positive associations indicated by closer than expected proximity in coordinate space and negative associations indicated by greater than expected distances in coordinate space. CXCL-8, interleukin 8; IL1β, interleukin 1β; IFNγ, interferon γ; TNFα, tumor necrosis factor α.

Co-inertia analysis of bacterial relative abundance and immune gene transcription also produced significant associations under both the control conditions (*R*^2^ = 0.25, *P* = 0.009) and in response challenge (*R*^2^ = 0.65, *P* = 0.001). The strongest associations were between *Flavobacterium spp*. and transcription of the genes *IL-1β, CXCL-8, and TNFα* (Fig. 4). Interestingly the positive associations exhibited under resting state conditions (Fig. 4A) become negative associations in response to challenge (Fig. 4B). *IFNγ* exhibited a comparatively weak negative association with *Flavobacterium spp*.

## Discussion

The changes in gene transcription at all assayed genes in response to the challenges in our study indicate that the genes we studied are responding to stress. Many of these genes have previously been demonstrated to respond to stress or immune challenge in other studies (e.g., Wiseman et al. 2007; Raida and Buchmann 2009). The different patterns of response and resting transcription we observed among populations suggest that diverging evolutionary processes (i.e., not stabilizing selection) are contributing to the variation among Babine Lake rainbow trout tributary populations. Population structure is present among Babine Lake rainbow trout tributary populations and neutral divergence in this system follows a pattern of isolation by distance (Koehler 2010); however, the lack of consistent clinal variation in gene transcription observed across a geographic gradient suggests, but does not preclude, that the differences are not a result of genetic drift. Local adaptation can occur when gene flow is reduced among populations and the environmental conditions experienced differ (Kawecki and Ebert 2004). The tributary populations we studied indeed experience variation in temperature fluctuations and extremes as well as variation in the composition of microbial communities, indicating that differential selection on gene transcription may explain the differences among populations. The results we present here are unlikely to reflect the fishes' previous environmental exposure given the acclimation period at the holding facility and known transcriptional dynamics of the genes that we studied (e.g., Raida and Buchmann 2009). The possibility exists that transcriptional patterns may reflect previous environmental exposure; however, additive genetic variation for transcriptional traits has been demonstrated (Brem and Kruglyak 2005; Roberge et al. 2007; Aykanat et al. 2012b) making it unlikely that all of the variation we observed was due to phenotypic plasticity or developmental programing. Furthermore, the potential for nonadditive genetic variation to contribute to adaptive phenotypes has been proposed (Aykanat et al. 2012a).

Transcriptional differences among natural salmonid populations have been demonstrated in the context of detrimental hybridization effects among locally adapted populations and aquaculture escapes (Normandeau et al. 2009; Tymchuk et al. 2010) as well as life history trade-offs among species pairs (St-Cyr et al. 2008). Our results are consistent with the conclusion that gene transcription profiles are population-specific, and hence may reflect local adaptation in the early rearing habitat of Babine Lake rainbow trout; however, phenotypic differences observed among isolated populations do not constitute strong evidence for local adaptation. To make a stronger case for local adaptation, we show that gene transcription is correlated with stream environments.

The range of temperatures recorded for our streams are consistent with those measured for other salmon-bearing streams (Elliot et al. 1998). Temperature ranges comparable to those we recorded have been investigated as drivers of selection on growth rates (Jonsson et al. 2001). Jonsson et al. (2001) failed to demonstrate a correlation between optimal growth temperature of juvenile Atlantic salmon and the thermal conditions of their streams; however, they suggested that differences in growth efficiency among populations might be linked, in part, to thermal conditions of the streams. Trade-offs between transcription of growth or survival (stress response) genes have been demonstrated among whitefish species pairs adapted to benthic and limnetic habitats (St-Cyr et al. 2008). In other species of fish, gene transcription-mediated adaptation to different temperature regimes has been demonstrated for metabolic genes, including *PK,* in *Fundulus heteroclitus* (Whitehead and Crawford 2006). Furthermore, experiments with wild-caught *Fundulus* have also demonstrated greater differences among populations at resting state than after a heat shock (Healy et al. 2010) consistent with our study. Perhaps the optimal strategy for coping with a stressful event is strongly selected upon and populations evolve to maintain different resting state transcription to balance energetic costs against the frequency of stressful events. To this end, trade-offs between transcription of growth and stress response genes have been demonstrated for both chronic and fluctuating heat stress in *Fundulus* (Podrabsky and Somero 2004) reinforcing the role trade-offs may play in the local adaptation of gene transcription.

Positive associations of maximum stream temperature with resting state transcription of genes controlling glycolysis (*PK*) are consistent with results from *Fundulus* (Whitehead and Crawford 2006) and suggest a role for increased metabolism of glucose in coping with thermal extremes. This interpretation is also consistent with the results of Wiseman et al. (2007) who demonstrated gene transcription patterns in the livers of rainbow trout that represent a reorganization of metabolism to facilitate the breakdown of energy-rich molecules and increased production of glucose for export to body tissues to cope with metabolic stress. The association of average daily temperature range with transcription of the rate-limiting enzyme for gluconeogenesis (*PEPCK*) indicates that experiencing larger fluctuations in temperature may require tissues to have a greater capacity to produce their own glucose. The negative associations of transcription in response to challenge may reflect an energetic trade-off where populations experiencing extremes more frequently have evolved to have a higher resting state transcription and thus require a reduced transcriptional response compared with those populations less frequently experiencing extremes. Until now, a link between stream temperature and local adaptation in salmonids had not been established (Garcia de Leaniz et al. 2007); however, it appears that temperature can play a role in modulating selection for the mobilization of glucose resources, which is likely to have an influence on growth and survival. Studies from *Fundulus* have demonstrated additional transcriptional differences among populations related to temperature regimes of heat-shock proteins (Fangue et al. 2006) and xenobiotic processing (Whitehead and Crawford 2006) which may be worthwhile investigating in future studies.

Despite our modest sample size for bacterial community analysis, we discovered a high level of diversity of microbial taxa in this system, consistent with marine studies utilizing massively parallel *16S* rRNA sequencing (e.g., Bolhuis and Stal 2011). Populations of fish from different streams are known to experience different microbial communities as these are often tied to bedrock geochemistry, water chemistry, temperature, and surrounding terrestrial ecosystems (e.g., Hullar et al. 2006). Furthermore, there is spatial diversity in the bacterial pathogens infecting juvenile salmonids (Dionne et al. 2009; Evans and Neff 2009). Among the genera detected in our study, we identified several as potentially pathogenic: *Flavobacterium psychrophilum* is the cause of cold-water disease and rainbow trout fry syndrome (Lorenzen et al. 1997), several members of *Pseudomonas* are opportunistic pathogens known to cause lesions and death in juvenile salmonids (e.g., Altinok et al. 2006) and *Mycobacterium* species have been implicated as the cause of fish disease (Arakawa and Fryer 1984). Many disease-causing bacteria in fish are opportunistic pathogens that become virulent during periods of stress (Harvell et al. 2002), suggesting that infection by previously un-described fish pathogens is also possible. The strength of selection resulting from pathogen pressure on juvenile salmonids is also inextricably linked to stream temperature because of the positive relationships between pathogen diversity, abundance, and temperature (Harvell et al. 2002; Dionne et al. 2009). However, it is likely that pathogen-mediated selection on juvenile salmonids would exceed that of temperature alone due to high mortality rates associated with disease outbreaks in young-of-the-year salmon (Holt et al. 1989). The positive association of multiple cytokine gene transcription and *Flavobacterium* relative abundance we demonstrated suggests a role for natural selection in determining population level differences in transcription. The inversion from positive to negative associations of cytokine genes following challenge may reflect the physiological pattern of expression for the cytokines we assayed which peak quickly and then fall as the immune response progresses (Raida and Buchmann 2009). The stronger correlation among immune gene transcription (*R*^2^ = 0.25, *P* = 0.009) versus metabolic gene transcription (*R*^2^ = 0.19, *P* = 0.013) and their relevant environmental parameters also indicates stronger selection imposed by pathogens on juvenile salmonids. Multiple lines of evidence for selection by specific pathogens as well as pathogen diversity on *MH* and other immune-related loci have been demonstrated for a variety of salmonid species (e.g., Dionne et al. 2007, 2009; Evans and Neff 2009; Tonteri et al. 2010; de Eyto et al. 2011). To our knowledge, our work represents the first evidence of local adaptation mediated by transcription of immune system candidate genes in natural populations.

We found *Flavobacterium spp*. to be positively associated with *IL-1β, CXCL-8,* and *TNFα* resting state gene transcription among populations of rainbow trout in Babine Lake, indicating that *Flavobacterium spp*. may be a potent selective agent in this system. One representative of this genus, *Flavobacterium psychrophilum*, is a cold-water pathogen that is most virulent at low temperatures (Holt et al. 1989). It causes lesions and can result in up to 90% mortality for rainbow trout fry. The positive association between transcription of cytokines and relative abundance of *Flavobacterium spp*. we demonstrated indicates that populations may be trading off the energetic costs of transcribing cytokines with the frequency of infections they experience. Higher levels of resting state transcription in certain populations may reflect the fish's ability or need to respond transcriptionally to infection. A reduced transcriptional response of cytokine genes to a secondary infection has been demonstrated for juvenile rainbow trout that survived a primary infection (Raida and Buchmann 2009). The reduced response Raida and Buchmann (2009) demonstrated was suggested to represent the development of adaptive immunity and a reduced reliance on innate (cytokine transcriptional) response. This would suggest that increased relative abundance of pathogens in the streams we studied results in a negative association with cytokine transcription due to the presence of acquired adaptive immunity. The absence of this pattern in our transcription data could be explained by the incomplete immunity of juvenile rainbow trout (Johnson et al. 1982) or by the diversity of other opportunistic pathogens experienced by juvenile rainbow trout in a complex natural environment.

In contrast to *Flavobacterium psychrophilum*, increases in the diversity and virulence of opportunistic pathogens are generally correlated with increasing temperature (Harvell et al. 2002). Despite this, little else is known about the specific pathogenicity and conditions favoring opportunistic infection by many other bacteria (McVicar et al. 2006). As more immunological studies are conducted under both laboratory and natural conditions, we will have a clearer picture of the potential threats previously un-described fish pathogens may pose for wild populations, as well as the dynamics of immune response in response to variable and complex natural environments (Pederson and Babayan 2011). A clear concern, as climates continue to warm, is the risk for more opportunistic infections to occur and create multiple stresses for fish species and populations already in decline (Crozier et al. 2008).

In conclusion, we provide evidence for the important role of gene transcription in mediating the process of local adaptation in tributary populations of rainbow trout. By providing a link between local environmental conditions and specific gene transcription profiles, we have strengthened the case that rapid evolution to local environments occurs, and have provided insight into the mechanisms that facilitate local adaptation of natural populations. Specifically, we highlight the role of temperature as a selective force on the transcriptome of salmonids both directly, by affecting the thermal regime fish experience, and indirectly, by influencing coexisting pathogen communities. We also provide the first evidence of local adaptation selection by pathogens on the transcription of immune-related genes. In light of climate change, the strength of selection by these direct and indirect means will undoubtedly change in unpredictable ways, likely leading to complex responses to local environmental variation. Finally, the population-specific response to stress we report reinforces the functional variability among genetically structured populations and emphasizes the need to conserve individual tributary populations to maintain maximal levels of genetic diversity and hence evolutionary potential.

## Data Accessibility

Raw and normalized gene transcription values have been uploaded to Dryad –doi:10.5061/dryad.662vr. Raw 454 sequence data will be uploaded to NCBI – SRA.

## Supporting Information

**Table S1.** Average population gene transcription values for candidate loci (Log_10_ Mean [SE]) among populations at resting state (Control) and average population transcriptional response to challenge (Log_10_ Mean Challenged – Log_10_ Mean Control [SE of difference]). Asterisks indicate significantly differentially transcribed genes in response to the challenges.
