# Supplementary material for: Environmental associations with gene transcription in Babine Lake rainbow trout: evidence for local adaptation
Source: Ecol Evol. 2013 Mar 19;3(5):1194–208. doi: 10.1002/ece3.531 (PMC3678475; doi:10.1002/ece3.531)
Supplement: Supplementary file 1 [file ece30003-1194-SD1.doc]

Table S1: Average population gene transcription values for candidate loci (Log_10_ Mean (SE)) among populations at resting state (Control) and average population transcriptional response to challenge (Log_10_ Mean Challenged – Log_10_ Mean Control (SE of difference)). Asterisks indicate significantly differentially transcribed genes in response to the challenges.

| Population | cathepsin D | GR | PEPCK | PK | IL-1β | CXCL-8 | IFNγ | TNFα |
| --- | --- | --- | --- | --- | --- | --- | --- | --- |
| *Control* |  |  |  |  |  |  |  |  |
| 11 Mile | -2.04 (0.09) | -2.00 (0.05) | -3.22 (0.16) | -3.38 (0.23) | -2.45 (0.12) | -3.84 (0.13) | -1.75 (0.10) | -3.32 (-0.08) |
| Tsak | -1.82 (0.11) | -1.68 (0.10) | -2.83 (0.16) | -2.91 (0.22) | -2.52 (0.06) | -4.32 (0.23) | -1.76 (0.16) | -2.98 (-0.12) |
| Tachek | -1.94 (0.14) | -1.94 (0.06) | -2.93 (0.08) | -3.50 (0.14) | -1.95 (0.16) | -2.83 (0.35) | -1.80 (0.10) | -2.50 (-0.25) |
| Cross | -1.93 (0.08) | -1.85 (0.08) | -3.22 (0.07) | -3.30 (0.10) | -2.34 (0.11) | -3.51 (0.22) | -1.46 (0.13) | -2.87 (-0.11) |
| Sutherland | -1.99 (0.07) | -1.84 (0.07) | -3.31 (0.16) | -3.63 (0.09) | -2.26 (0.14) | -4.08 (0.11) | -1.70 (0.21) | -3.00 (-0.23) |
| Duncan | -2.76 (0.26) | -1.86 (0.02) | -2.60 (0.30) | -3.60 (0.11) | -2.36 (0.04) | -3.92 (0.06) | -1.67 (0.03) | -3.11 (-0.07) |
| *Challenged* |  |  |  |  |  |  |  |  |
| 11 Mile | 0.33 (0.11)* | 0.43 (0.05)*** | 0.34 (0.16) | 0.22 (0.23) | 0.25 (0.13) | 0.22 (0.14) | 0.14 (0.11) | 0.30 (0.09)* |
| Tsak | 0.27 (0.11)* | 0.05 (0.11) | -0.21 (0.17) | -0.37 (0.23) | 0.47 (0.07)*** | 0.54 (0.25) | 0.10 (0.17) | 0.03 (0.13) |
| Tachek | 0.06 (0.21) | 0.31 (0.08)** | 0.27 (0.11)* | -0.09 (0.31) | -0.49 (0.20)* | -1.09 (0.40)* | -0.50 (0.19)* | -0.70 (0.26)* |
| Cross | 0.19 (0.09) | 0.22 (0.10) | 0.21 (0.13) | 0.21 (0.18) | -0.20 (0.12) | -0.42 (0.24) | -0.45 (0.15)* | -0.40 (0.15)* |
| Sutherland | 0.34 (0.09)** | 0.14 (0.08) | 0.30 (0.20) | 0.50 (0.22)* | -0.41 (0.15)* | -0.12 (0.14) | -0.44 (0.22) | -0.39 (0.24) |
| Duncan | 1.08 (0.26)** | 0.14 (0.06)* | -0.46 (0.3) | 0.23 (0.13) | -0.31 (0.07)** | -0.15 (0.09) | -0.40 (0.09)*** | -0.25 (0.12) |

* = P < 0.05, ** = P < 0.01, *** = P < 0.001
